# Growth of broiler chickens, and physical features of the digestive system, and leg bones after aluminosilicates used

**DOI:** 10.1038/s41598-022-25003-w

**Published:** 2022-11-28

**Authors:** Jakub Biesek, Mirosław Banaszak, Kamil Kądziołka, Sebastian Wlaźlak, Marek Adamski

**Affiliations:** Department of Animal Breeding and Nutrition, Faculty of Animal Breeding and Biology, Bydgoszcz University of Science and Technology, Mazowiecka 28, 85-084 Bydgoszcz, Poland

**Keywords:** Zoology, Animal physiology

## Abstract

The assessment of aluminosilicates’ impact on the production of chickens, the physical features of the intestines, and leg bones was done. 500 Ross 308 chickens were used and divided into 5 groups. The control group was I. Groups II, III, IV, and V were fed with halloysite and zeolite (1:3 ratio) at 0,5% (1–35 days; starter, grower 1 and 2) and 1% (36–42 days; finisher) levels. Aluminosilicates were also used for the peat litter: II—500 g of halloysite/m^2^; III—250 g of halloysite/m^2^ and 250 g of zeolite/m^2^; IV—500 g of zeolite/m^2^; V—130 g halloysite/m^2^, 370 g zeolite/m^2^. During 42 days, growth and feed indicators were recorded. 10 birds from each group were selected for slaughter. The digestive tract, femur, and tibia bones were sampled, and physical features were analyzed (weight, length, and strength). A lower feed conversion ratio on days 23–35 was found in the groups with the aluminosilicates addition. In group V a lower weight of the gizzard was found than in group I. A liver weight was higher in group V than in group III. A higher strength of the femurs was demonstrated in group IV. The tibia bones were characterized by higher strength than the femurs of broiler chickens. The aluminosilicates to feed and litter had no adverse effect.

## Introduction

Poultry farming is a dynamically developing production in the agri-food industry^1^. The world’s largest poultry meat producers are the USA, China, and Brazil. In European Union, Poland is a leader. According to the data presented, in 2020, 2.18 million tons of carcass weight of broiler chickens were produced in Poland, which constitutes 19.53% of the total EU production^2^. The greatest threat to the supply–demand balance in the poultry market is posed by outbreaks of avian influenza throughout the country that limit the export of products to other countries, as well as the SARS-COV-2 coronavirus pandemic, which limited the demand and fluctuation of meat supply chains in the HoReCa sector^3^.

Poultry producers face many challenges regarding effective production, good quality raw materials, and maintaining a balance with the natural environment. However, one should also look at the safety of meat production technology and the proper functioning of broiler chickens. A properly developed digestive system should characterize broiler chickens to ensure the appropriate functioning of the body during intensive production. The proper functioning of the digestive tract enables high utilization of the feed ingredients and production efficiency^4^. It plays an essential role in metabolic and digestive processes and is responsible for the absorption of nutrients. It is also crucial in the process of the body's immune response^5^. The rapid increase in muscle weight of broiler chickens increases the susceptibility to bone injuries. It is associated with considerable economic losses for producers because skeletal structure defects negatively affect birds' welfare and even cause deaths^6^. Durable leg bones, including the femoral and tibial ones, enable movement and facilitate the uptake of feed and water, consequently achieving appropriate weight gain and bird health^7^. After the ban on antibiotic growth promoters (AGP) in poultry nutrition, the search for alternatives in the form of feed additives that have a beneficial effect on the health of the kept birds, the safety of animal products, and the natural environment began^8^.

The aluminosilicates, as an additive, can be added to feed and bedding. It could be an optimal solution in poultry production. Research has shown that these additives can affect production performance, carcass characteristics, and bird welfare by reducing the occurrence of *footpad dermatitis* (FPD)^9^. According to the EU regulation, feed additives are substances, microorganisms, or preparations other than feed material and premixtures that are intentionally added to feed or water to perform one or more functions. Feed additives must benefit the feed’s properties and positively impact the environment when used in production. Feed additives also increase animal welfare, mainly due to the impact on the gastrointestinal microbiota and the digestibility of feed^10^. One of the feed additives classified as zootechnical (technological) additives is aluminosilicates. These minerals have high absorption values, increasing birds’ performance and overall health by showing no significant absorption and removal of nutrients and vitamins. However, they bind toxins in feed and transport them through the digestive tract without harming the animal organism^8^. Adding aluminosilicates to the feed may slow down the digestive processes, extending the retention time of the food content, and stimulating digestion and absorption of nutrients, which improves efficiency^11^. The aluminosilicates were also used as a litter additive as an odor and ammonia reduction agent^12^.

A beneficial effect was also noted when using aluminosilicates on intestinal morphology. Natural clinoptilolite positively affects gut parameters, including acting on microbial populations of the digestive tract^13^. In the available literature, mainly the feed supplement in the form of a probiotic was analyzed on morphometric features of intestines in chicken broilers. The studies showed no effect of the addition of probiotics on the length and diameter of individual sections of the intestine and the weight of the proventriculus, gizzard, and spleen. A shorter small intestine characterizes commercial hybrids with high genetic potential for intensive production compared to broilers reared in extensive rearing^14,15^.

Bone strength depends on the following factors: genotype, sex, age, nutrition, anti-nutritional substances, hormones (steroid hormones, testosterone), and the presence of disease^16^. Lameness and osteoporosis negatively affect the welfare of birds, making it difficult to move, which leads to problems with the correct intake of feed and water and increased mortality in the flock^17^. One of the threats to the skeletal system is its low endurance due to the rapid weight gain of broiler chickens. Nutrition plays a crucial role in the rearing of chickens, which significantly impacts bone strength. In the available literature, the following physical parameters of bones were determined: strength, stiffness, thickness^18^, length, weight, width, and the Seedor index (bone mass/length) were also analyzed^19^. Well-developed bone and skeletal system of chickens highly affects the higher technological usefulness of carcasses (processing). It is essential for ensuring high-quality products of animal origin, like poultry meat^20^. Strength is a crucial indicator of bone susceptibility to fractures and the course of the bone mineralization process^18^.

The intensification of production while maintaining appropriate standards of the obtained poultry meat requires optimization of rearing conditions, including feeding, and ensuring a suitable environment, including bedding. Using zeolite and halloysite has potential as both a zootechnical additive for feed and bedding. Adding minerals can enhance the functioning of the digestive system and its development (intestinal endurance) and reduce diseases related to the skeletal system of chickens, including leg bones. Obtaining carcasses from chickens whose intestinal walls are characterized by a high level of strength is important from the processing point of view (biosafety when gutting carcasses).

The presented research results are part of the "Safe Farm" project. The study revealed whether the aluminosilicates do not reduce the strength of the intestines and bones in the safety of post-slaughter processing aspect. Therefore, research was undertaken to assess the effect of the natural zootechnical additive in the form of aluminosilicates on broiler chickens' production results, the digestive system's physical features, and leg bones. The research hypothesis is that adding aluminosilicates to feed and bedding impacts the production results and physical characteristics of the digestive system and leg bones of Ross 308 broiler chickens.

## Materials and methods

The research and rearing were carried out following applicable law.

### Experiment design and chicken rearing

500 1-day-old male Ross 308 broiler chickens were used in the study. The birds were kept in environmental conditions by the recommendations of Ross 308 broiler chickens production (Aviagen), whit some modifications. The rearing was carried out in cooperation with the poultry farm, which was included in the project. The temperature was 30 °C on the day of insertion, 27 °C on day 7, and 21 °C on day 21, and to the end of rearing. Air humidity was 60–65%, and ventilation was 1 m^3^/kg body weight/hour. The lighting intensity was 20 lx. Chickens had provided with six 10-min dark periods during the first week, followed by 18 h of light and 6 h of darkness on the 7th day. In the last three days before slaughter, continuous lighting was provided in the poultry house. The birds were kept on peat litter. The peat litter was characterized by a dry weight of 95.07%, a pH of 6.04, and a nitrogen content of 3.55% (average values obtained in laboratory analyses for control purposes). The rearing period lasted 42 days. The birds were divided into five equally numerous groups, kept in 1 × 1 m experimental pens, where the mesh was made of stainless material. Each group was maintained in ten replicates (10 chickens each). The feed and water were administered ad libitum. Complete feed was commercial, and their composition complied with the feeding standards for broiler chickens according to the nutritional recommendations^21^. The feeding was divided into four periods, i.e., starter feed from the 1st to the 10th day of rearing, grower 1 from the 11th to the 22nd day of rearing, grower 2 from the 23rd to the 35th day of rearing, and the finisher type feed from the 36th to the 42nd the day of rearing. Control group (I) was fed based on the complete feed without additives. Experimental groups (II, III, IV, V) were provided with complete feed with the addition of halloysite and zeolite in the ratio of 1:3. In the first three feeding periods, the addition of aluminosilicates was 5 g/1 kg of feed. In the last stage (finisher), the addition was increased to 10 g/1 kg of feed. In the experimental groups, aluminosilicates were also used for the litter in a loose form (added on top). In group II, 500 g of halloysite were added per 1 m^2^ of peat surface; in group III—the addition of halloysite was 250 g/m^2^ of peat and zeolite 250 g/m^2^ of peat, and in group IV—the addition of zeolite 500 g/m^2^ of peat, while in group V—addition of halloysite 130 g/m^2^ of peat and zeolite 370 g/m^2^ of peat. The characteristics and chemical composition of halloysite and zeolite are presented in Table 1. The data in the table are obtained from the supplier of the aluminosilicates.

**Table 1:** Characteristics and chemical composition of halloysite and zeolite (supplier information).

Zeolite		Halloysite	
Specific surface	30–60 m^2^/g	Specific surface	65–85 m^2^/g
Bulk volume	1.60–1.80 kg/m^3^	Bulk volume	0.70–0.85 g/cm^3^
Weight	2.20–2.44 kg/m^3^		

%		%	
SiO_2_ (silicon dioxide)	71.30	Al (aluminum)	13.00
Al_2_O_3_ (aluminum oxide)	13.20	Si (silicon)	12.00
CaO (calcium oxide)	5.20	Ca (calcium)	0.40
K_2_O (potassium oxide)	3.40	Mg (magnesium)	0.30
Fe_2_O_3_ (iron (III) oxide)	1.90	Na (sodium)	0.10
MgO (magnesium oxide)	1.20	K (potassium)	0.08
Na_2_O (sodium dioxide)	1.30	P (phosphorus)	0.30
TiO_2_ (titanium dioxide)	0.30	Fe (iron)	9.00
Si/Al (silicon/aluminium)	5.40	Ti (titanium)	1.00
%		Mn (manganese)	0.20
Clinoptilolite	84.00		
Cristobalite	8.00		
Mica clay	4.00		
Plagioclase	3.50		
Rutile	0.20		

### Growth performance

During the rearing period, the body weight (g) of broiler chickens (BW) was monitored. On the day of chicks insertion (1), feed changes (11, 23, 36) and on the day of slaughter (42), birds were individually weighted (Radwag, Radom Polska). The feed intake (FI) was recorded daily. Based on the collected data, the body weight gain (BWG) was calculated using the formula: BWG (g) = bird body weight on the last day of the feeding period (g) − bird body weight on the first day of the feeding period (g). The feed conversion ratio per 1 kg of body weight gain (FCR) was also calculated, from the formula $FCR (\frac{\mathrm{kg}}{\mathrm{kg}})=\frac{FI (\mathrm{kg})}{BWG (\mathrm{kg})}$. The production results were calculated for individual feeding periods and the entire rearing period. After rearing, the condition of the soles of the feet of broiler chickens was assessed. The measurement method was done according to Bilgili et al.^22^, where the 3-point visual ranking system was used (0, footpads with no lesions present; 1, mild lesions, and dermal ridges with oval or round ulcers covered with a crust; 2, severe lesions, dark brown crust).

### Samples collection

After 42 days of rearing, the birds were weighed (Radwag, Radom, Poland), and based on the averages obtained within each pen, broiler chickens were selected with a body weight similar to the group’s mean value. The birds fasted for 8 h before slaughter. The slaughter was performed by breaking the spinal cord (cutting between the first vertebra of the cervical segment and the occipital condyle—decapitation) after stunning the birds. A total of 50 chickens (10 from each group) were slaughtered. The carcasses were scalded in hot water at 65 °C for 10 s, then the feathers were removed, and the ankle legs were cut off. To examine gutting, the digestive tract and organs were dissected and placed in distilled water in sterile containers. The carcasses and the prepared samples were cooled for 24 h in a refrigerator (Hendi, Poznań, Poland) at a temperature of 4 °C. After 24 h, the carcasses were subjected to a simplified dissection to collect the femurs and tibia. The legs at the hip joint were cut off, and the bones were dissected for further analysis. Additionally, based on the live body weight and carcass weight, slaughter yield was calculated, using formula:

$$slaughter\, yield\, \left({\%}\right)=\frac{carcass \,weight\, (g)}{live\, body \,weight \,(g)}\times 100{\%}.$$

### Physical features of the digestive system and leg bones

The digestive system was drained of water (the digestive tract was dried after being removed from containers with water, and the remaining intestinal content was squeezed out), and morphometric measurements were made. The digestive system was weighed (empty). The digestive tract has been divided into individual sections: duodenum, jejunum, ileum, cecum, and colon. The proventriculus, the gizzard, and the liver and pancreas were distinguished. All elements were weighed (Radwag, Radom, Poland), and their length was measured using a measuring tape (cm). Based on the collected data, the relative weight and length of individual sections of the digestive system (to body weight) were calculated. Methods were done similarly as Stęczny and Kokoszyński^15^ reported.

Each part of the small and large intestine was subjected to tensile strength analysis. The test strength analysis was performed using the Instron 3345 device (Instron, Buckinghamshire, UK). The Bluehill 3.0 software (3345, 2013) was used. The tensile strength properties of the intestines were performed by evaluating the maximum force at break (N). The intestinal load was simulated using the PNEUMATIC GRIP 2KN attachment. Standardized samples (equal to 10 cm in length) were positioned between two attachments and stretched. The measurement rate was 500 mm/min. The tensile strength of the intestines was performed according to the method described by Biesek et al.^23^. As with the intestines, the breaking strength of the femurs and tibias was analyzed using the BEND FUTURE 10 mm ANVIL adapter. The bones were placed between the grips, and the maximum load and force at fracture (N) and compression strain were measured. The measurement rate was 250 mm/min. The methods were described by Kuźniacka et al.^24^.

### Statistical calculation

The collected data were compiled in a statistical program (Statistica, 13.3, Statsoft, TIBCO, Cracow, Poland). The mean values for each tested feature were calculated concerning the experimental factor (groups I–V). The standard error of the mean (SEM) was calculated for all groups. The distribution was normal (Shapiro–Wilk test). One-way analysis of variance was used. Statistically significant differences between the groups were verified using Tukey's post-hoc test. The *P*-value is assumed to be less than 0.05.

## Results

### Growth performance

Table 2 shows the production results of broiler chickens for each feeding period and the entire rearing period. On day 10, the BW was significantly higher in group I compared to V (*P* = 0.020), while on day 22, the chickens from group I were statistically significantly heavier than in groups II, IV, and V (*P* < 0.001). However, the final BW (on day 42 of rearing) did not differ significantly between the groups (p = 0.592). Body weight gain (BWG) in group I was significantly higher than in group V on days 1–10 and 11–22 (*P* = 0.015; *P* < 0.001, respectively). On the other hand, on days 23–35, significantly lower BWG was demonstrated in group I than in groups II and IV (*P* = 0.001). On days 36–42, the BWG was higher than group III (*P* = 0.024). However, for the entire rearing period (days 1–42), no statistically significant differences in the BWG values were found (*P* = 0.594). Broiler chickens in groups I and II were higher FI on days 1–10 than in groups IV and V (*P* < 0.001). Feed intake on days 23–35 was higher in group I than in group V (*P* = 0.033). Analyzing the results of feed consumption per 1 kg of body weight gain (FCR), it was found that in group II FCR was significantly higher than in group IV in the first feeding period (days 1–10, *P* = 0.004). On days 11–22, FCR was significantly higher in groups II, IV, and V compared to the control group (*P* = 0.002). A significant increase in FCR in group I, compared to groups II, IV, and V, was found on days 23–35 (*P* < 0.001). No statistically significant differences for FI and FCR on days 36–42 and for the whole rearing period (days 1–42) were found (*P* > 0.05). After slaughter, significantly higher slaughter yield in groups III and IV (> 76%) compared to I and II (< 75%) was found (*P* = 0.042).

**Table 2:** Growth performance of broiler chickens.

Item	Group^A^					SEM	*P*-value
	I	II	III	IV	V		
**BW (g)**							
1st day	44.01	44.86	45.38	44.72	45.58	0.81	0.054
10th day	373.78^a^	364.11^ab^	371.54^ab^	354.59^ab^	349.43^b^	2.85	0.020
22nd day	1123.57^a^	1017.34^b^	1049.46^ab^	1037.47^b^	1005.12^b^	9.87	< 0.001
35th day	2346.72	2409.71	2384.97	2404.28	2324.31	17.62	0.483
42nd day	2944.37	3099.34	3088.35	3087.98	2959.70	23.22	0.592
**BWG (g)**							
Days 1–10	329.77^a^	319.25^ab^	326.16^ab^	309.87^ab^	303.85^b^	2.86	0.015
Days 11–22	749.79^a^	653.23^b^	677.92^b^	682.87^ab^	655.69^b^	8.30	< 0.001
Days 23–35	1223.15^b^	1392.38^a^	1335.50^ab^	1366.82^a^	1319.19^ab^	14.53	0.001
Days 36–42	597.65^b^	689.62^ab^	703.39^a^	683.69^ab^	635.39^ab^	12.11	0.024
Days 1–42	2900.36	3054.48	3042.97	3043.26	2914.12	23.19	0.594
**FI (g/bird)**							
Days 1–10	361.20^a^	354.00^a^	338.60^ab^	320.00^b^	324.80^b^	3.49	< 0.001
Days 11–22	888.60	873.00	871.53	898.27	855.90	5.11	0.076
Days 23–35	2580.33^a^	2489.82^ab^	2555.70^ab^	2495.82^ab^	2413.40^b^	19.39	0.033
days 36–42	997.21	1083.91	1061.05	1053.60	1003.32	10.70	0.053
Days 1–42	4840.38	4930.60	4981.20	4794.56	4616.02	45.24	0.091
**FCR (kg/kg)**							
Days 1–10	1.10^ab^	1.11^a^	1.04^ab^	1.03^b^	1.07^ab^	0.01	0.004
Days 11–22	1.19^b^	1.34^a^	1.28^ab^	1.32^a^	1.31^a^	0.01	0.002
Days 23–35	2.11^a^	1.79^b^	1.93^ab^	1.83^b^	1.83^b^	0.03	< 0.001
Days 36–42	1.67	1.60	1.53	1.54	1.64	0.04	0.681
Days 1–42	1.67	1.61	1.63	1.57	1.59	0.02	0.358
Slaughter yield (%)	74.89^b^	74.90^b^	76.58^a^	76.03^a^	75.83^ab^	0.32	0.042
**Footpad dermatitis (%)**							
Score 0	98	99	100	99	100		
Score 1	2	1	0	1	0		
Score 2	0	0	0	0	0		

*BW* body weight, *BWG* bodyweight gain, *FI* feed intake, *FCR* feed conversion ratio, *SEM* standard error of the mean.

^a,b^Values with different letters in a row differ significantly, *P* < 0.05.

^A^Groups II, III, IV, V were fed with halloysite and zeolite (1:3 ratio) at 0,5% (1–35 days) and 1% (36–42 days) level. Aluminosilicates were also used for the litter: II—500 g of halloysite/m^2^; III—250 g of halloysite/m^2^ and 250 g of zeolite/m^2^; IV—500 g of zeolite/m^2^; V—130 g of halloysite/m^2^, 370 g zeolite/m^2^.

When analyzing the skin lesions of the soles of the feet, no *footpad dermatitis* (FPD) was found. It was shown that the cases assessed according to the scale, with a score of 1, did not exceed 1% of the assessed groups. The score of 2 was not present. In group I, 2% of birds had small changes on the soles that indicated FPD, and in groups II and IV–1%.

### Physical features of the digestive tract and leg bones

The results of the weight and length of individual parts of the digestive system per 1 kg of body weight of broiler chickens are shown in Table 3. A significantly higher weight of the gizzard was found in group I than in group V (*P* = 0.043). The weight of the liver was significantly higher in group V than in group III (*P* = 0.037). There were no significant differences in the tensile strength of individual sections of the digestive system (*P* > 0.05). Only quantitatively higher strength of the duodenum and jejunum in all experimental groups (II-V). The ileum in groups II and III, the cecum in groups II, III, and IV, and the colon in groups III, IV, and V were found to be higher in tensile strength than in group I (Table 4). The significantly higher breaking strength of the femur bones in group IV than in group I, expressed as the maximum load value (*P* = 0.021) and load at breaking (*P* = 0.013), was noticed. When analyzing the breaking strength of the tibia, no statistically significant differences were found between the groups (*P* > 0.05). Comparing the femurs with the tibia bones, a statistically significantly lower breaking strength of the femurs, expressed in the maximum load (*P* < 0.001) and lower load at fracture (*P* < 0.001), was demonstrated. Comparing each segment of the digestive system, there was a significantly higher tensile strength of the colon compared to the duodenum and both the rest of the parts, i.e., jejunum, ileum, and caecum (*P* < 0.001) (Table 5).

**Table 3:** Relative weight and length of individual sections of the digestive system of broiler chickens.

Item	Group^A^					SEM	*P*-value
	I	II	III	IV	V		
**Weight (g/kg body weight)**							
Digestive system (empty)	34.18	32.86	32.23	31.12	32.78	3.87	0.829
Proventiculus	4.16	4.30	5.03	4.08	4.17	0.18	0.474
Gizzard	11.40^a^	9.54^ab^	8.81^ab^	9.03^ab^	8.43^b^	2.40	0.043
Liver	20.48^ab^	20.39^ab^	19.29^b^	20.17^ab^	20.92^a^	1.21	0.037
Pancreas	2.96	2.88	3.20	2.43	2.42	0.12	0.192
Duodenum	6.49	5.47	5.47	5.52	5.75	0.19	0.421
Jejunum	11.47	12.00	11.91	11.02	11.52	0.32	0.903
Ileum	10.33	8.9	9.28	8.94	9.99	0.38	0.721
Cecum	4.05	4.42	3.85	3.99	3.82	0.14	0.695
Colon	1.83	2.08	1.71	1.65	1.71	0.06	0.157
**Length (cm/kg body weight)**							
Digestive system (empty)	86.37	80.55	80.77	80.65	81.25	7.60	0.738
Duodenum	13.80	11.50	12.01	12.00	11.99	0.27	0.054
Jejunum	30.95	28.17	28.65	29.37	30.09	0.71	0.775
Ileum	30.91	31.25	30.17	29.71	29.23	0.85	0.952
Cecum	6.82	6.13	6.38	5.89	6.47	0.14	0.256
Colon	3.89	3.50	3.56	3.71	3.48	0.08	0.391

*SEM *standard error of the mean.

^a,b^Values with different letters in a row differ significantly, *P* < 0.05.

^A^Groups II, III, IV, V were fed with halloysite and zeolite (1:3 ratio) at 0,5% (1–35 days) and 1% (36–42 days) level. Aluminosilicates were also used for the litter: II—500 g of halloysite/m^2^; III—250 g of halloysite/m^2^ and 250 g of zeolite/m^2^; IV—500 g of zeolite/m^2^; V – 130 g halloysite/m^2^, 370 g zeolite/m^2^.

**Table 4:** Tensile strength of individual sections of the digestive system of broiler chickens.

	Group^A^					SEM	*P*-value
	I	II	III	IV	V		
**Duodenum**							
Maximum force (N)	4.90	6.42	5.36	5.62	6.19	0.37	0.731
Displacement (mm)	40.04	28.85	34.04	19.68	31.86	3.29	0.411
**Jejunum**							
Maximum force (N)	2.84	3.57	3.83	3.61	3.40	0.20	0.610
Displacement (mm)	37.94	34.76	33.01	32.50	32.13	1.49	0.757
**Ileum**							
Maximum force (N)	3.45	3.89	3.55	3.09	3.16	0.20	0.761
Displacement (mm)	38.87	38.49	38.41	33.43	33.33	1.27	0.427
**Caecum**							
Maximum force (N)	3.38	3.54	4.39	4.32	3.05	0.22	0.211
Displacement (mm)	31.06	28.41	34.45	24.59	29.77	1.44	0.294
**Colon**							
Maximum force (N)	6.05	5.65	8.29	8.90	7.06	0.46	0.107
Displacement (mm)	27.29	30.67	25.34	26.85	22.71	1.95	0.806

	Duodenum	Jejunum	Ileum	Caecum	Colon		
Maximum force (N)	6.17^b^	3.78^c^	3.55^c^	4.15^c^	7.99^a^	0.20	< 0.001
Displacement (mm)	42.13	47.10	44.88	43.94	31.64	1.97	0.111

*SEM* standard error of the mean.

^a,b^Values with different letters in a row differ significantly, *P* < 0.05.

^A^Groups II, III, IV, V were fed with halloysite and zeolite (1:3 ratio) at 0,5% (1–35 days) and 1% (36–42 days) level. Aluminosilicates were also used for the litter: II—500 g of halloysite/m^2^; III—250 g of halloysite/m^2^ and 250 g of zeolite/m^2^; IV—500 g of zeolite/m^2^; V—130 g of halloysite/m^2^, 370 g zeolite/m^2^.

**Table 5:** Breaking strength of femur and tibia bones of broiler chickens.

Item	Group^A^					SEM	*P*-value
	I	II	III	IV	V		
**Femur bone**							
Maximum load (N/mm)	307.56^b^	404.55^ab^	418.00^ab^	430.62^a^	340.86^ab^	102.10	0.021
Load at fracture (standard, N)	297.34^b^	388.05^ab^	407.16^ab^	430.32^a^	310.79^ab^	109.37	0.013
Strain under compression at fracture (mm/mm)	0.05	0.06	0.05	0.05	0.08	0.04	0.600
**Tibia bone**							
Maximum load (N/mm)	279.65	267.35	309.09	280.93	261.18	10.71	0.684
Load at fracture (standard, N)	263.57	248.30	294.58	269.82	261.18	11.96	0.817
Strain under compression at fracture (mm/mm)	0.05	0.04	0.04	0.05	0.05	0.02	0.925

	Femur bone	Tibia bone					
Maximum load (N/mm)	271.95^b^	380.32^a^	10.38	< 0.001			
Load at fracture (standard, N)	260.13^b^	366.73^a^	11.08	< 0.001			
Strain under compression at fracture (mm/mm)	0.05	0.06	0.003	0.057			

*SEM* standard error of the mean.

^a,b^Values with different letters in a row differ significantly, *P* < 0.05.

^A^Groups II, III, IV, V were fed with halloysite and zeolite (1:3 ratio) at 0,5% (1–35 days) and 1% (36–42 days) level. Aluminosilicates were also used for the litter: II—500 g of halloysite/m^2^; III—250 g of halloysite/m^2^ and 250 g of zeolite/m^2^; IV—500 g of zeolite/m^2^; V—130 g halloysite/m^2^, 370 g zeolite/m^2^.

## Discussion

In the studies of Qu et al.^25^, a zeolite addition to the feed was applied at 10 g/kg of feed. The authors showed an increase in daily body weight gain (BWG) over 42 days and higher feed intake (FI) and BWG from day 1 to day 21 of rearing. Raj et al.^26^ showed that the addition of a mycotoxin-reducing substance with modified zeolite in feed for broiler chickens at the level of 1 g/kg and 2 g/kg improved the feed conversion ratio (FCR). In the study by Banaszak et al.^27^, broiler chickens were reared with the addition of aluminosilicates in the feed at the level of 0.5–2% (25% halloysite and 75% zeolite) in the feed and 0.950 kg/m^2^ of wheat litter (1:1 halloysite and zeolite) were characterized by higher BWG without affecting FI and FCR. Using zeolite and halloysite 0.5–2% in the feed, with the simultaneous addition of aluminosilicates to the rye litter at 800 g/m^2^ (with different proportions), resulted in higher BW and BWG of broiler chickens. However, FI increased in some groups^28^. In rearing chickens, the aluminosilicates mentioned above were used in various configurations, also for bedding in the form of wheat pellets^29^. Higher BW and partially lower FCR were shown in the chicken group, where zeolite and halloysite were used for feed and bedding. Hcini et al.^30^ also tested the effect of a feed addition of zeolite in turkeys. Zeolite was added at the level of 1 and 2%. The beneficial effect of aluminosilicate on production performance and higher BWG was found. Most of the cited authors stated that adding zeolite or halloysite to the litter had a positive or no adverse effect on the production results of broiler chickens. However, a 4% share of zeolite in the feed harmed BWG and increased the FCR of Cherry Valley and Orvia broiler ducks of both sexes^23^. The use of halloysite for a feed at 0.5% and 500 g per 1 m^2^ of bedding area did not harm production results. At the same time, chickens had a lower presence of *footpad dermatitis* and better bedding quality^31^. In our study, no significant influence of experimental factor on the dermal lesions on feet could be explained by suitable quality litter, where the moisture was approximately—5%.

Shariatmadari^32^ presented an overview of research on the use of zeolite in poultry production. The differences in both favorable and unfavorable results may be due to the different proportions of zeolite in the feed. This can affect the concentration of various nutrients, the consistency of the feed, and the production performance. Analyzing the results of our research and the cited authors, no negative impact of the aluminosilicates addition in rearing poultry was found at the level of up to 2%. Too high aluminosilicate addition may negatively affect the adsorption of nutrients and coccidiostats. In addition, the origin of the minerals, their type (including zeolite, bentonite, and kaolin), and the composition of complete mixtures should be considered^33^.

Wawrzyniak et al.^34^ described that the zeolite addition in the feed for broiler chickens plays a significant role in the final stage of digestion and nutrient assimilation, which results in improved weight gain, feed conversion ratio, and growth rate of the small intestine. The research found higher BWG at individual rearing stages. However, the weight and length of individual intestinal sections were similar, regardless of the presence of aluminosilicates in the feed. As in our research, Khadem et al.^35^ showed a lower gizzard weight with zeolite. According to the cited authors, gizzard weight may be related to the level of microbial contamination of the feed. In the own research, no analyzes were performed regarding the presence of microorganisms in the feed material. Gizzard development is also related to its stimulation by maintaining the digesta^36^. Referring to the cited authors' description, it can be concluded that aluminosilicates affect the shorter passage time of the feed through the gastrointestinal tract without adversely affecting the production results.

Minerals (including aluminosilicates) are insoluble, and their properties can improve intestinal peristalsis and reduce feed density. According to researchers, feed additives may increase the endurance of the digestive system^37–39^. In our research, no statistically significant differences were found between the groups in the tensile strength of individual sections of the digestive system (small and large intestine). However, the experimental groups were characterized by quantitatively higher values. From the point of view of industry (processing), it is a crucial element of poultry production to ensure that the condition of the digestive system, including intestine tensile strength, is at an appropriate level. Lower-strength intestines may pose a microbiological hazard when gutting carcasses on the production line^40^. Contamination of poultry carcasses with *Salmonella* and *Campylobacter* bacteria most often occurs when the digestive tract is interrupted, and the intestinal contents enter the surface of the meat^41,42^.

The strength of the bones is important from the point of view of optimizing the welfare of kept chickens for slaughter and further processing poultry carcasses and meat. Bone strength depends on the proper growth of birds and the availability of calcium and phosphorus from the feed^43^. Depending on their mineralization, physical characteristics, including mechanical bone fracture strength, are modified by birds feeding^44^. Pirzado et al.^45^ used aluminosilicates (azomite) at a level of 0.25% compound feed for broiler chickens. The authors showed that the addition of a natural mineral increased bone mineralization and the digestibility of calcium and phosphorus from the feed, which resulted in higher bone-breaking strength. The studies showed that the femurs were characterized by higher breaking strength in all groups (quantitatively), while significantly higher values were obtained only in group IV. Despite the same addition of aluminosilicates to the feed-in groups II, III, IV, and V, the addition to the bedding was different (IV—zeolite only), which could enhance the effect. This relationship cannot be confirmed in the case of the tibia-breaking strength analysis. Shariatmadari^32^ described that the use of zeolite in poultry production has a positive effect on bone strength. The aluminosilicates contain calcium compounds and phosphorus particles (Table 1), which could positively affect bone-breaking strength.

The addition of zeolite and halloysite to the feed in the ratio of 75:25 at the level of 0.5–1% and to the litter in various proportions, in the amount of up to 500 g per 1 m^2^ of bedding area did not adversely affect the production results throughout the rearing period. The beneficial effect of mineral supplements was found on days 23–35, where aluminosilicates significantly decreased the feed conversion ratio. Added aluminosilicates to feed (halloysite 25:zeolite 75, 0.5–1%) and to the litter (130 g/m^2^ of halloysite and 370 g/m^2^ of zeolite) resulted in a lower weight of the gizzard. There was a beneficial effect on the slaughter yield in group III—250 g of halloysite/m^2^ and 250 g of zeolite/m^2^ of litter, and IV—500 g of zeolite/m^2^ of litter. No significant effect of zeolite and halloysite was found on the relative weight and length of individual intestinal sections and their tensile strength. The addition of aluminosilicates to feed (halloysite 25:zeolite 75, 0.5–1%) and to the litter (500 g/m^2^ of zeolite) had a positive effect on the breaking strength of the femurs. The tibia bones were characterized by higher breaking strength than the femurs of broiler chickens. The addition of 0.5–1% of aluminosilicates to feed in a ratio of 1:3 (halloysite:zeolite) and to the bedding at the level of up to 500 g/m^2^ in various proportions had no negative impact on production results and the development of the digestive system and physical properties of bones. It is a critical aspect of the welfare, biosafety of production, and further technological processing of raw materials from poultry.

### Ethics

The research was done with recommendations of directive no. 2010/63/EU and resolution 13/2016 of the National Ethics Committee for Animal Experiments of June 17, 2016 (marking animals does not require the consent). The approval of the Ethics Committee was not required. The experiment was carried out following the applicable regulations. The slaughter of the birds was carried out following the relevant regulations of animal handling during slaughter, including humane treatment. The methods used in meat quality testing were also carried out under the current and commonly used methodology described in the Material and methods section. According to directive no. 2010/63/EU of 22 September 2010 on the protection of animals used for scientific purposes, the consent of the Ethics Committee was not required. The directive sets out requirements for the protection of animals used for experimental purposes. It is described that these rules do not apply to agricultural activities and animal husbandry. The experiment was carried out in conditions similar to commercial ones, so the farm owners were responsible for the production. Slaughter for tissues and organs collected from animals is not a procedure (Act of January 15, 2015, on protecting animals used for scientific or educational purposes, item 266, Journal of Laws of the Republic of Poland).

According to the regulations, the consent of the ethical committee for this type of scientific activity is not required. Slaughter was performed following procedures, and the removal of tissues (carcasses, digestive system, bones) after slaughter is not a procedure that requires the approval of the ethics committee. If the animals used in the agricultural research are kept under similar commercial conditions, the slaughter is performed following Annex I to Council Regulation (EC) No 1099/2009 of 24 September 2009 on the protection of animals at the time of the killing.

In the described experiment, the stunning birds (by Table 3 of Annex I. Concussion / hit with a truncheon to the head—applies to birds up to 5 kg), and decapitation were performed.

Slaughter for removing tissues (carcass, digestive system and bones) is not a procedure that requires the ethics committee’s approval.

Scientific activities in the manuscript were recorded and reported in annual reports, obligatorily submitted to the Animal Welfare Team of the Department of Animal Breeding and Biology Bydgoszcz University of Science and Technology. It is in accordance with the ARRIVE guidelines.

## Data Availability

The datasets analyzed during the current study are available from the corresponding author on reasonable request. If there are some questions, the authors remain at your disposal.
